# A Multi-Scale Modeling Framework for Individualized, Spatiotemporal Prediction of Drug Effects and Toxicological Risk

**DOI:** 10.3389/fphar.2012.00204

**Published:** 2013-01-22

**Authors:** Juan G. Diaz Ochoa, Joachim Bucher, Alexandre R. R. Péry, José M. Zaldivar Comenges, Jens Niklas, Klaus Mauch

**Affiliations:** ^1^Insilico Biotechnology AGStuttgart, Germany; ^2^Unité Modèles pour l’Écotoxicologie et la ToxicologieINERIS, Verneuil-en-Halatte, France; ^3^ECVAM, Institute for Health and Consumer Protection, European Commission Joint Research CentreIspra, Italy

**Keywords:** acetaminophen, toxicity testing, pharmacokinetics, drug metabolism, hepatotoxicity

## Abstract

In this study, we focus on a novel multi-scale modeling approach for spatiotemporal prediction of the distribution of substances and resulting hepatotoxicity by combining cellular models, a 2D liver model, and whole body model. As a case study, we focused on predicting human hepatotoxicity upon treatment with acetaminophen based on *in vitro* toxicity data and potential inter-individual variability in gene expression and enzyme activities. By aggregating mechanistic, genome-based *in silico* cells to a novel 2D liver model and eventually to a whole body model, we predicted pharmacokinetic properties, metabolism, and the onset of hepatotoxicity in an *in silico* patient. Depending on the concentration of acetaminophen in the liver and the accumulation of toxic metabolites, cell integrity in the liver as a function of space and time as well as changes in the elimination rate of substances were estimated. We show that the variations in elimination rates also influence the distribution of acetaminophen and its metabolites in the whole body. Our results are in agreement with experimental results. What is more, the integrated model also predicted variations in drug toxicity depending on alterations of metabolic enzyme activities. Variations in enzyme activity, in turn, reflect genetic characteristics or diseases of individuals. In conclusion, this framework presents an important basis for efficiently integrating inter-individual variability data into models, paving the way for personalized or stratified predictions of drug toxicity and efficacy.

## Introduction

The need to develop a virtual physiological human for clinical and pharmacological applications has stimulated the development of several physiological models that capture the interplay between different structures in tissues, organs, and the whole body (Fenner et al., 2008). The liver is an important organ in this workflow because it is required for many metabolic functions. This fact has boosted the development of virtual liver models within the framework of large-scale research programs. Prominent examples are the virtual liver project (Wambaugh and Shah, 2010) and the virtual liver network (Holzhütter et al., 2012). In a number of liver modeling efforts, relatively simple cells were coupled to models of liver tissue to perform qualitative predictions of substance distributions and cell responses, particularly toxicity. In some pharmacokinetic models, data obtained from *in vitro* experiments are translated into different transporter and enzyme activities, which are thereafter distributed in the liver (Pang et al., 2007). Other approaches defined complete *in silico* livers where the organ is coupled with a simple model of cell metabolism. For instance, Hunt and Ropella (2008) and Wambaugh and Shah (2010) not only made a comprehensive model for simple cells with a simple metabolism but also developed a model that allows an estimation of the substance distribution in the lobule assuming that its structure resembles a network where each hepatocyte is located in each node of the network. Based on this description, the spatial distribution of the substance can be reproduced from the portal to the central vein. The advantage of such approaches is the possibility of predicting substance extraction and distribution also depending on the heterogeneity of the liver micro structures (Ropella and Hunt, 2010). In several organ models, relatively simple individual cells are coupled to a complex description of the liver (Kuepfer et al., 2012). For the prediction of function and structure of the liver, such coarse grained approaches provide essential information on the physics (behavior of a granular media), the way the liver responds to damages, and on the detoxification and drug elimination of this organ (Chelminiak et al., 2006). Examples are models where cell populations are described as multi-agent systems ordered in complex networks of the parenchymal tissue (Chelminiak et al., 2006; Hoehme et al., 2010). However, a detailed description of the metabolic and regulatory networks is necessary for understanding the liver function, in particular for the prediction of the effects of drugs (and other substances) in pharmaceutical research (Kuepfer et al., 2012). In this field, only a few models have recently taken steps toward the integration of detailed cell mechanisms (Ohno et al., 2008). For instance, there are changes in the distribution of oxygen and metabolites inside the liver introducing a zonation that affects the function (Allen et al., 2005) as well as cell death in response to toxic doses (Malhi et al., 2010). An additional advantage of the incorporation of detailed dynamic cellular models is the possibility to include inter-subject variability in predictions of drug effects (Bucher et al., 2011; Niklas et al., 2012).

In this work, we further developed multidimensional models for the liver which were coupled to *in silico* cells performing a metabolic function. The primary goals of this study were (i) to set-up and verify a whole body model coupled with an *in silico* liver, (ii), to predict the distribution of substances for an *in silico* patient treated with acetaminophen, and (iii) to extrapolate critical doses from *in vitro* data. One of the main goals of this approach is to simulate cell mortality when acute toxicity takes place. To this end, we reconstructed a network for acetaminophen metabolism, integrated this into an *in silico* liver model, simulated uptake and distribution of drug and metabolites in the liver and the whole body of an *in silico* patient, and performed simulations upon administration of different single doses.

## Materials and Methods

### Modeling of acetaminophen metabolism and toxicity

The metabolic network model for metabolism and toxicity of Acetaminophen (APAP) was set-up based on literature data. In brief, acetaminophen is metabolized by cytochrome P450 monooxygenases (CYPs; Patten et al., 1993; Thummel et al., 1993; Chen et al., 1998), UDP-glucuronosyltransferases (UGTs; Court et al., 2001; Mutlib et al., 2006; Riches et al., 2009), and sulfotransferases (SULTs; Sweeny and Reinke, 1988; Adjei et al., 2008; Riches et al., 2009). Glutathione (GSH)-transferases (GSTs; Coles et al., 1988) contribute additionally to Phase II conjugation. APAP is degraded mainly to the corresponding glucuronide (APAPG) and sulfate (APAPS) metabolites and by CYP-mediated oxidation to *N*-acetyl-*p*-benzoquinone imine (NAPQI; Chen et al., 2008). Amongst CYPs, several isoenzymes contribute to NAPQI formation in liver, with probable major contributions by CYP3A4, CYP2E1, CYP2A6, and CYP1A2 (Patten et al., 1993; Thummel et al., 1993; Chen et al., 1998) and minor contribution by CYP2D6 (Dong et al., 2000). The toxic metabolite NAPQI is detoxified through conjugation with GSH by GSTs to APAPGS (Coles et al., 1988). Additionally, NAPQI can be reduced back to APAP by NADPH quinonereductase (NQO1; Moffit et al., 2007). Furthermore, intracellular unspecific binding of APAP and metabolites to proteins and lipids was considered, particularly for APAPS and NAPQI due to their comparably low fraction unbound. Binding constants were calculated using logP-correlations (Zaldivar Comenges et al., 2011).

APAP is taken up into the cell via active transport and by passive diffusion (McPhail et al., 1993). Since NAPQI is similar to APAP and comparably lipophilic with respect to the logP (PubChem), passive diffusion is also assumed for NAPQI. Permeability coefficients were estimated using logP-correlations (Zaldivar Comenges et al., 2011). APAP-Glucuronide and APAP-Sulfate are excreted through the transporter multidrug resistance related protein (MRP)2 and translocated at the basolateral side via MRP3/4 (Xiong et al., 2000, 2002; Chen et al., 2003; Zamek-Gliszczynski et al., 2006). APAPGS is also exported via MRP2 and MRP3/4 (Chen et al., 2003; Zamek-Gliszczynski et al., 2006).

At low APAP dose, intracellular NAPQI concentration is very low since it is immediately conjugated with GSH. GSH is replenished by glutathione synthase (GSS; Reed et al., 2008) and degraded via gamma-glutamyltransferase (GGT; Shaw and Newman, 1979). At over dose conditions, NAPQI accumulates and initiates severe hepatoxicity (Rumack and Matthew, 1975). The cellular toxicity pathway of APAP includes increased NAPQI-binding to mitochondrial membrane proteins (James et al., 2003) triggering perturbation of the respiration machinery and inducing increased formation of reactive oxygen (ROS) and nitrogen (RNS) species (Jaeschke et al., 2003).

ROS are detoxified in mitochondria by superoxide dismutase and glutathione peroxidase (GPX; Murphy, 2009). Therefore, the cellular GSH level is further reduced through increased H_2_O_2_ synthesis resulting in increased formation of oxidized glutathione disulfide (GSSG). GSSG is reduced back to GSH by glutathione reductase (GSR). ROS and RNS cause lipid peroxidation and protein nitration (Jaeschke et al., 2003; James et al., 2003), respectively. Both mechanisms finally destroy mitochondrial integrity.

**Figure 1:**
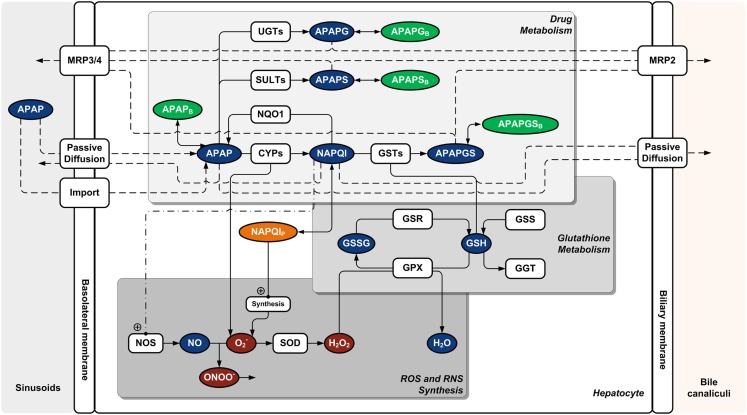
**Cellular metabolic network model for acetaminophen metabolism and toxicity**. Abbreviations: APAP, acetaminophen; APAPG, acetaminophen glucoronide; APAPGS, acetaminophen-glutathione-conjugate; APAPS, acetaminophen sulfate; CYP, cytochrome P450 monooxygenase; GGT, γ-glutamyltransferase; GPX, glutathione peroxidase; GSH, glutathione; GSR, glutathione reductase; GSS, glutathione synthase; GSSG, glutathione disulfide; GST, glutathione *S*-transferase; MRP (2/3/4), multidrug resistance related protein; NAPQI, *N*-acetyl-*p*-benzoquinone imine; NOS, nitric oxide synthase; NQO1, NADPH quinonereductase; SOD, superoxide dismutase; SULT, sulfotransferase; UGT, UDP-glucuronosyltransferase. Index “B,” non-specifically bound (protein/lipid); index “P,” non-specifically bound to protein.

Accordingly, a cellular kinetic metabolic model of APAP-metabolism, GSH turnover, ROS synthesis, and cell death was set-up (Figure 1). The mathematical model is described in a supplementary material.

The probability of cell death (necrosis) is defined to be a function of the concentration of substances in the cell triggering the deterioration of the hepatocyte, which is similar to the approach suggested by Wambaugh and Shah (2010). The production of H_2_O_2_ after ROS synthesis and the consumption of GSH are both used as criteria for cell death in our liver model. If the H_2_O_2_ concentration surpasses a critical value and GSH is consumed, the probability of necrosis for the hepatocytes increases. Furthermore, since the cells in the centrilobular region have a low oxygen intake (Allen et al., 2005), we assume that the cells in the respective region are more sensitive to toxic effects compared with cells near the portal veins. This means that the probability of cell death via necrosis is inversely proportional to the distance to the central vein (Gujral, 2002).

### Liver and multi-scale model: Structure of sinusoids and lobules

**Figure 2:**
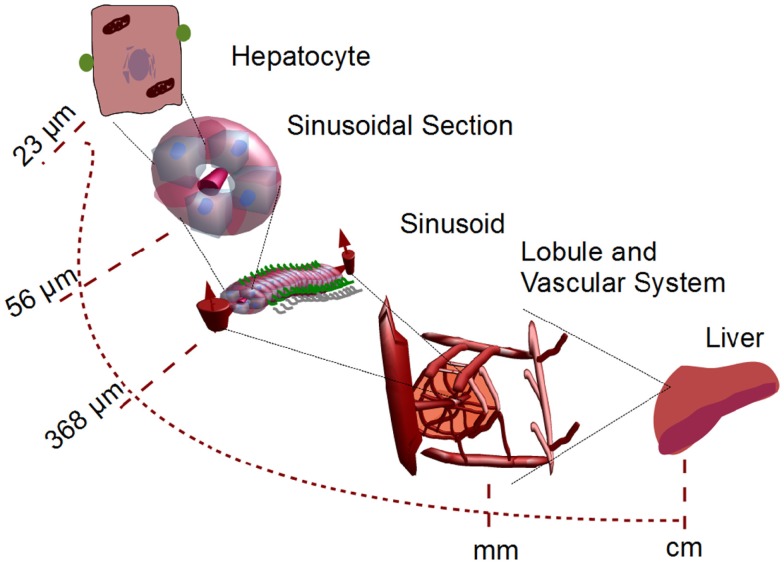
**Multi-scale system from single hepatocytes to organ level**. Single hepatocytes are coupled to liver capillaries (sinusoids) which are coupled to micro-organelles called lobules. These lobules are considered to be the smallest functional micro-structure in the liver. The corresponding parameters for the lobule module are shown in Table 1.

**Figure 3:**
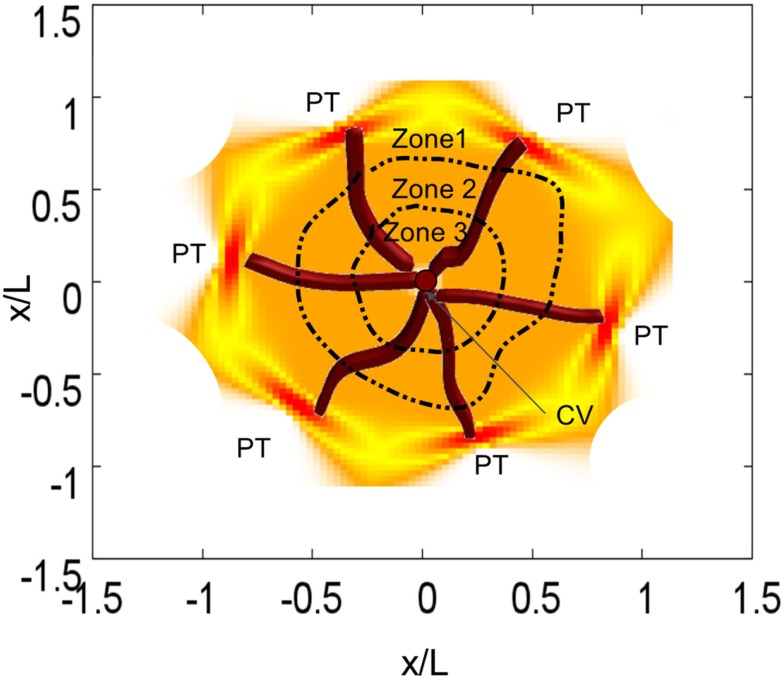
**Representation of one lobule with six sinusoids**. Each sinusoid transports blood form a portal triad (PT) to the central vein (CV). We also included lobule zonation.

The liver is a complex organ with several interconnected structures across several scales (Figure 2). A central part of our modeling approach is integration of (i) metabolism of single hepatocytes, (ii) transport of substances across the lobules, and (iii) the whole body. After the blood enters the liver, it is distributed by portal veins into functional subunits, called lobules, which carry out diverse functions including the detoxification of xenobiotics at cell level (Figure 1). An adult liver contains around one million lobules, distributed more or less homogeneously across the liver (Arias et al., 2009). In the periphery of each lobule, several blood vessels deliver the blood flow into the lobule through additional substructures called sinusoids. We simplify this structure by assuming a parallel tube model. With this assumption, the lobule is represented as a hexagonal structure with six portal triads, each connected to a simple tube draining the blood from portal to central vein (Pang and Rowland, 1977; Figure 3).

The sinusoid is essentially modeled as a collection of hepatocytes aligned along a blood vessel with geometry similar to a cylindrical tube (Ohno et al., 2008). Since the central goal of this study was to model drug metabolism in the liver taking place in the hepatocytes, we define the sinusoids as simple capillary structures with a wall composed only by hepatocytes. We assume that four hepatocytes are arranged in each section of the tube. For each section, there is a concentration of compounds *c_m_*(*t*, *x*) where m is an index for the corresponding compound, *x* is the position of the cell on the sinusoid (*L* is the total length of the sinusoid), and *t* is the time. Substance concentrations as a function of time *t* and hepatocyte position along the sinosoid *x* is obtained by solving the corresponding coupled differential equation.

(1) $$\frac{\partial c\left(x,t\right)}{\partial t}=\text{N}\cdot \text{v}\left(\text{c}\left(x,t\right),p\right),$$

where ***c***(*x*, *t*) is a *m* dimensional vector of intracellular and extracellular concentrations [i.e., ***c***(*x*, *t*) = *c*_1_ (*x*, *t*), *c*_2_(*x*, *t*),…, *c_m_*(*x*, *t*)], ***N*** is the *m* × *r* stochiometric matrix, ***v*** is the *r* dimensional vector of reaction rates that depends on the substrate concentrations *c* and a set of parameters *p*.

We approximate the geometry of the sinusoid to a tube with an average radius of 5 μm where the substance is dispersed on the *x* axis. Further assuming that the blood is a good solvent, the distribution of the substance can be described as an advection-diffusion process, which is described by the following equation.

(2) $$\frac{\partial c_{sinus}}{\partial t}+U_{x}\frac{\partial c_{sinus}}{\partial x}=D\frac{\partial^{2}c_{sinus}}{\partial x^{2}};$$

Here, *U_x_* is the velocity of the suspension along the tube and *D* the diffusion coefficient. We assume that the average blood velocity in the sinusoid is about 0.1 mm/s (Vollmar and Menger, 2009). Additionally, we take the Fahraeus effect into account, i.e., the tendency of red cells to migrate away from the tube wall so that the mean velocity of the cells is larger than the mean velocity of the suspension near the cell wall. Sugihara-Seki and Fu (2005) found that the mean velocity of the suspension *U_x_* is about 0.05 mm/s. Additionally, we assume that the blood in the sinusoid is a liquid with a low Reynolds number (<0.0001)so that the Einstein (1905) relation can be employed, i.e.,

(3) $$D=\frac{K_{\text{B}}T}{f\pi \eta R_{\text{g}}},$$

where *D* is the diffusion constant, *K*_B_ is the Boltzman constant, *T* is the temperature and *f* is a number characterizing the boundary conditions of the particle. In this case, it is valid to assume sticky boundary conditions, i.e., that the fluid has a zero velocity relative to the surface of the molecule, assuming that this molecule can be approached by a sphere such that *f* = 6 (Cappelezzo et al., 2007). Additionally, η is the viscosity of the blood (which is about 3 × 10^−3^Pa × s, see, e.g., Késmárky et al., 2008, and *R*_g_ is the radius of gyration. Using the radius of gyration for acetaminophen (2.99 Å), the diffusion coefficient is about 2.22 × 10^−10^ m^2^/s (Falk, et al,. 2004).

The solution of Eq. 2 is

(4) $$c_{sinus}\left(t,x\right)=\frac{Q}{\pi r^{2}\sqrt{4D\pi t}}e^{-\frac{{\left(x-U_{x}t\right)}^{2}}{4Dt}},$$

with *Q* being the quantity of the substance (i.e., *Q* = *c* × *V*, *V*, is the volume), and *r* the radius of the sinusoid. The initial condition in this propagation is the concentration entering from the portal vein multiplied by the fraction unbound, *fu* (in this case *fu* = 0.75; Péry et al., 2012). We also assume that the liquid, distributed according to *c*_sinus_(*t*, *x*), propagates in discrete steps of time (similar to Wambaugh and Shah, 2010). With such an approach, the complex dynamics of the dispersion of the substance is significantly reduced without losing information on the hepatic elimination/metabolism of the substance. Simultaneously, each hepatocyte can transport, eliminate, and transform the substance in the bulk so that each chemical species has its own spatial distribution. The constants used for the modeling of the lobule are resumed in Table 1. The corresponding constants for individual cells as well as for the transport cell/bulk are assigned in the model for cell metabolism described above (see also the Supplementary material).

**Table 1:** **Physical parameters of lobule and sinusoids**.

Parameter	Symbol	Value	Reference
Blood velocity	*U_x_*	0.1 mm/s	Vollmar and Menger (2009)
Hepatocyte size	*l*_h_	23 μm	Hoehme et al. (2010)
Sinusoid diameter	*d*_s_	10 μm	Arias et al. (2009)
Number of sinusoids per lobule	*n*_s_	6	Wambaugh and Shah (2010)
Number of hepatocytes along the sinusoid	*n*_h_	16	Hoehme et al. (2010)
Diameter lobule	*d*_L_	1 mm	Arias et al. (2009)
Fraction unbound (APAP)	*f*_u_	0.75	Ishii et al. (2002)

The oxygen gradient along the sinusoid (see Modeling of Acetaminophen Metabolism and Toxicity) introduces a zonation in the liver, which, in turn, induces a heterogeneous distribution of CYP expression factors radially aligned to the lobule (Allen et al., 2005; Figure 3). This zonation is not only relevant in the physiology of the liver but also in the construction of *in silico* livers (Ropella and Hunt, 2010). Based on different evidence from the literature (Bhatia et al., 1999; Allen et al., 2004, 2005) we assume three regions with different CYP activity. Zone 2 is close to the periportal region whereas zone 3 is close to the pericentral region. We assume that the CYP3A4 activity is similar in zones 1 and 2; in zone 3 this value is about 1.3 times larger than in zone 1 (Oinonen and Lindros, 1998).

### Whole body model

**Figure 4:**
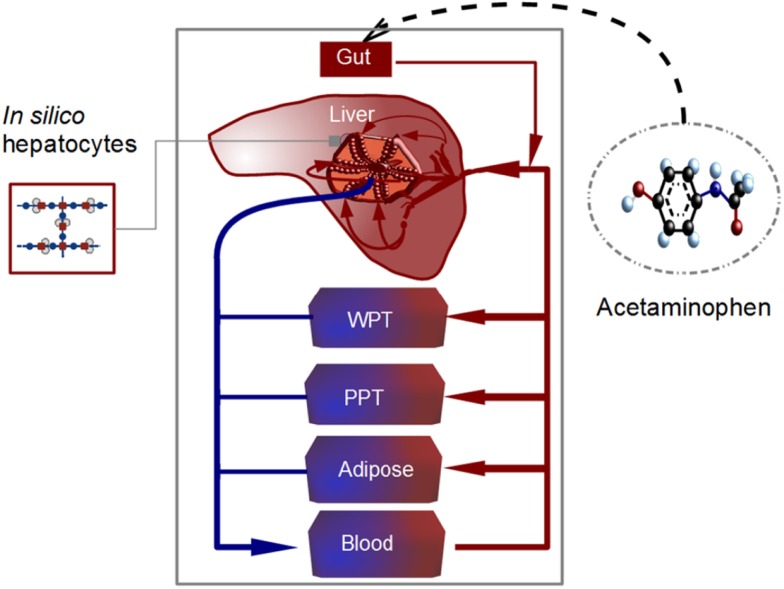
**Scheme of the whole body model**. The model is divided into liver, adipose tissue, blood, other well-perfused tissues (WPT), and other poorly perfused tissues (PPT). Blood transporting acetaminophen flows through the *in silico* liver, consisting of lobules coupled to *in silico* hepatocytes (containing the metabolic network represented in Figure 1). Acetaminophen is orally administrated and transported from gut to liver. The degradation and metabolization of the substance takes place in the hepatocytes adjacent to and alongside the sinusoids.

The estimation of a realistic microdosimetry requires the computation of the substance distribution in the whole body. For such a mathematical representation of the whole body, individual organs not reconstructed by an internal structure are represented as compartments neglecting concentration gradients of substances within the organ (Figure 4). The modular structure of the set-up allows for replacing the compartments by detailed structured organs, as required. In our model, we assume that metabolism of APAP is primarily performed by the liver. Consequently, there is no detailed representation for lung, kidney, or other organs. In this study, the whole body model consists of well-perfused tissues (WPT), poorly perfused tissues (PPT), adipose tissue, blood, and a structured 2D liver model. Finally, following the anatomical structure of the body, these compartments are interconnected through the blood circulation.

Our model is calibrated for an adult weighing 73 kg (Table 2). The tissue volumes were scaled from body weight and the blood flows scaled from cardiac output with the values used by Mielke and Gundert-Remy (2009). WPT as well as PPT can be represented as a perfusion rate-limited, one compartment model as follows [in the following, *C_i_*(*t*) are concentrations in the whole body, and *c_m_*(*t*) concentrations at the cell level].

(5) $$V_{i}\frac{dC_{i}\left(t\right)}{dt}=F_{i}\left(C_{b}\left(t\right)-\frac{C_{i}\left(t\right)}{PC_{i}}\right),$$

**Table 2:** **Parameters in the PBPK model depicted in Figure 4 according to Price et al. (2003)**.

	Organ	Blood flow (l/min)	Volume (l)	Partition coefficients (PC*_i_*)
1	Adipose tissue	0.56	28.0	0.25
2	Liver	1.3	1.82	0.774
3	WPT	3.5	5.68	0.774
4	PPT	1.63	35	0.66
5	Blood	6.4	5.7	0.774
6	Plasma	6.4	3.4	0.774

*The partition coefficients were defined according to Rodgers and Rowland (2006). WPT, other well-perfused tissues; PPT, other poorly perfused tissues*.

Where *i* is an index enumerating the organs (1 to 6 according to Table 2) with volume *V_i_*. *F_i_* is the blood flow in the corresponding organ, *C_i_*(*t*) is the concentration of the substances (APAP) in the organ, *C*_b_(*t*) = *C*_5_(*t*) is the concentration of the substance in blood and PC*_i_* is the corresponding partition coefficient of the organ. We assume that the substance distributes into major tissue constituents (water, bound to proteins, neutral lipids, phospholipids) and that there is a total unbound concentration in plasma of 0.75.

In our model, the absorption rate of APAP from stomach to gut is given by the following equation.

(6) $$\frac{dQ_{gut}\left(t\right)}{dt}=Fr_{ing}\cdot Q_{adm}\left(t\right)-k_{gut}\cdot Q_{gut}\left(t\right).$$

In this equation, *Q*_adm_(*t*) = Weight·Dose (*t*; weight given in kg, dose in mg/kg) is the amount of substance that is administrated and *Q*_gut_(*t*) = *C*_gutVgut_ is the amount of acetaminophen in the gut. The fraction of ingested APAP entering the liver, Fr_ing_, is fixed to 0.9 (Brown et al., 1979) and *k*_gut_, the rate of adsorption from gut to stomach, is fixed to 0.025 min^−1^(Péry et al., 2012).

We constructed organelles in the liver, coupled these to the cell metabolism, and made an estimation of the liver clearance, which is the capacity of the liver to eliminate and transform compounds from the blood. We simulate the sinusoid coupled to the hepatocyte dynamics and estimate the amount of APAP that the sinusoid/liver is able to eliminate, i.e., $E_{k}\left(t\right)=\left[\Sigma_{x=1}^{L}c_{\text{APAP}-cyt}\left(t,x\right)-c_{\text{APAP}-B}\left(t,L\right)\right],$ where *E_k_*(*t*) is the acetaminophen eliminated by the sinusoid *k* (*k* between 1 to *n*_s_, the total number of sinusoids), *c*_APAP − cyt_ is the concentration of acetaminophen in the cell, *c*_APAP − B_ is the concentration of acetaminophen in the sinusoidal bulk, and *L* is the length of the sinusoid [see a similar definition of clearance by Wambaugh and Shah (2010)]. The liver clearance is therefore given by $\frac{\Sigma_{k=1}^{n_{s}}{E_{k}}_{\left(t\right)}}{n_{s}}.$ The equation for the liver coupled to the whole body model (which is equivalent to the construction of a sub-compartment for the liver) is

(7) $$V_{\text{liv}}\frac{dC_{\text{liv}}\left(t\right)}{dt}=F_{\text{liv}}\left(C_{a}\left(t\right)-\frac{C_{\text{liv}}}{\text{P}{\text{C}}_{2}}\right)+k_{gut}\cdot C_{gut}-\xi \frac{\sum_{k=1}^{n_{s}}E_{k}\left(t\right)}{n_{s}}.$$

where ξ is a constant that couples the estimated clearance with the liver compartment, which is related to the parenchymal volume. Given that the parenchymal tissue is smaller than the total liver volume (about 80%; Arias et al., 2009), we set ξ = 0.75 in order to adjust the clearance of the lobule to the total volume of the liver; *PC*_2_ corresponds to the partition coefficient of the liver (see Table 2). Note that the rate of assimilation is an average value obtained from the integration in the lobule and that our liver is equivalent to a mean lobule.

Finally, the urinary excretion rate of acetaminophen was based on the estimate for rats through biometric scaling and was fixed to 0.016 l/min (Péry et al., 2012).

### Model verification

**Figure 5:**
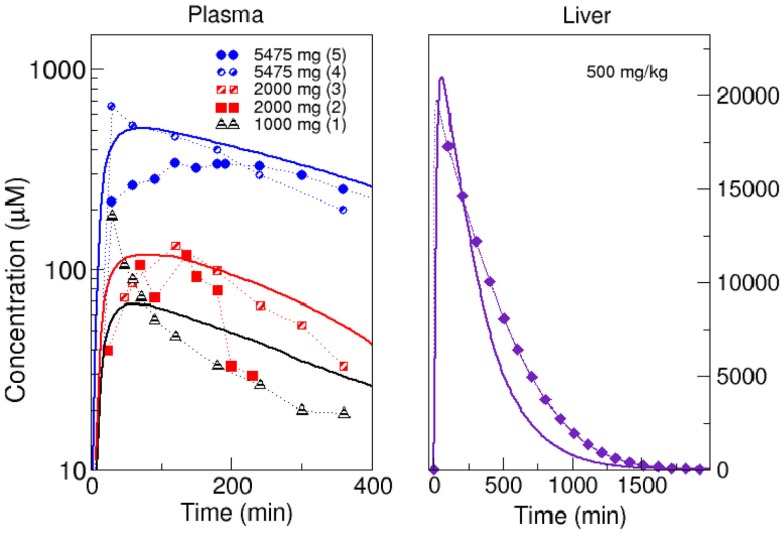
**Comparison of model simulations with experimental data**. Left panel: simulated acetaminophen concentration in plasma (solid lines) compared with*in vivo* pharmacokinetics: (1) single dose of 1000 mg (Rawlins et al., 1977), (2) single dose of 2000 mg (Rawlins et al., 1977), (3) single dose of 2000 mg (Brunner et al., 2002), (4) single dose of 5475 mg (Douglas et al., 1996), and (5) single dose of 5475 mg (Ly et al., 2004). Right panel: comparison of model simulation (solid line) with estimated concentration of acetaminophen in the liver using a PBPK model (Péry et al., 2012) after administration of a single dose of 500 mg/kg.

Before starting simulations, we verified the whole body model using experimental results for the concentration of acetaminophen in blood plasma after a single dose. We compared our predictions with results from literature for three different doses. For doses of 1000 mg (Rawlins et al., 1977), 2000 mg (Rawlins et al., 1977; Brunner et al., 2002), and 5475 mg (Douglas et al., 1996; Ly et al., 2004), the χ^2^ values were 0.71, 0.91, and 0.2, respectively, which indicates a relatively good fit of our model with experimental data. We also compared the result obtained with our whole body coupled to the *in silico* liver with a PBPK model coupled to a well-stirred liver for the uptake of acetaminophen in humans (Péry et al., 2012). A good agreement between the time courses (χ^2^ = 4.22) was observed (Figure 5). The deviations between our model and literature may originate from differences between the parameterization of the model (physiological parameters, enzyme activities) and the physiology of the individuals.

### Modeling and simulation platform

The metabolic network was generated by the software *Insilico* Discovery™ (*Insilico* Biotechnology AG, Stuttgart, Germany) and represented in FORTRAN. Once the individual cells are coupled to the sinusoid, the six sinusoids are simulated in parallel together with the whole body model. Numerical integration was performed using the LIMEX integrator for differential-algebraic equations (Nowak et al., 1996).

Specific enzyme activities were calculated from activities measured on recombinant enzymes (which are given in nmol_Drug_/nmol_Enzyme_/min) applying the following physiological parameters: Microsomal CYP content (Shimada et al., 1994), total cell protein (30 g/L_cell_, L_cell_ = 1 l of cells; Bucher et al., 2011), and ratio of microsomal to total cell protein (0.22; Bucher et al., 2011). Because we use cellular systems for kinetic modeling, we have to calculate concentrations (μmol/L_cell_) and activities (μmol/L_cell_/min) per cell volume.

## Results

### Spatiotemporal prediction of drug and metabolite concentrations

**Figure 6:**
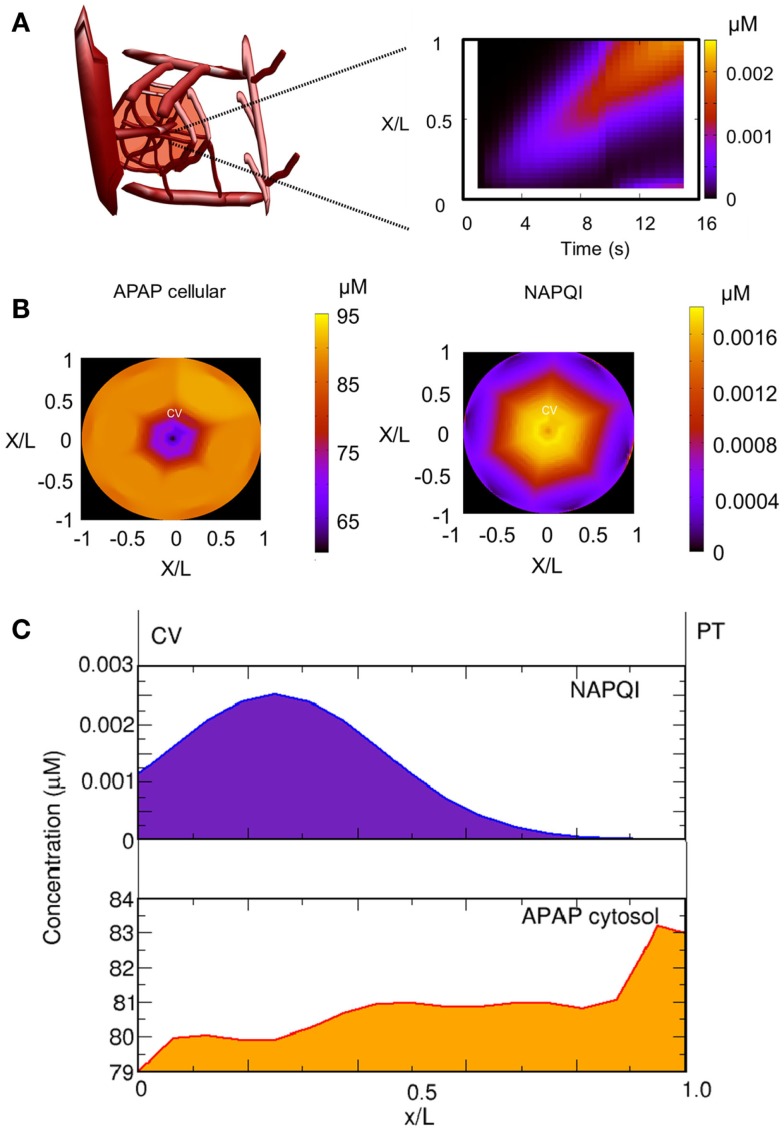
**Spatial distribution of acetaminophen (APAP) and NAPQI**. **(A)** Spatial distribution of APAP in the sinusoid after 10 min for a single dose of 393 mg/kg. The distribution of NAPQI is schematically given as a function of the distance (sinusoidal length normalized as *x*/L) and time. **(B)** Distribution of cellular APAP and NAPQI in the lobule at 4 h after a single APAP dose (393 mg/kg). **(C)** Mean distribution of NAPQI and cellular APAP in the lobule as a function of the sinusoidal length.

One advantage of our approach is the possibility to follow distributions of substances along liver lobules, which allows a detailed analysis of the accumulation of toxic substances in the organ. According to our model, APAP is distributed from the portal to the central vein showing a rapid transport of acetaminophen in the periportal region. Accordingly, toxic substances should accumulate in the pericentral region. This case can be verified by analyzing the accumulation of NAPQI (Figure 6), which can be used as a marker for APAP toxicity. We observed that NAPQI concentration (Figures 6B,C) were highest in the pericentral region indicating that the cells in this region should be more prone to APAP toxicity.

### Prediction of *in vivo* toxicity by combining *in vitro* data and the multi-scale model

A central aim of this study is to estimate cell viability and not just concentrations in the organ. To this end, we used experimental results of cell viability obtained from *in vitro* experiments (Zaldivar Comenges et al., 2011; Péry et al., 2012). In these experiments, a LC_50_(concentration at which 50% of the cell population are viable) of ∼4000 μM was observed. We performed simulations for *in silico* cells and defined that the critical concentration of H_2_O_2_ inducing cell death is the one obtained at the LC_50_ of APAP in the *in vitro* setting. We observed that intracellular H_2_O_2_ concentrations above 4000 μM can be used as an indicator for cell toxicity and assume that this threshold value is the criterion triggering cell death, i.e., if the concentration of H_2_O_2_ in the cell is higher than this critical value, necrosis takes place, meaning that intracellular functions stop or, in other words, that *dc*(*x*, *t*)/*dt* = 0. In some studies, it was shown that the necrosis process upon exposure to high H_2_O_2_ concentrations is relatively fast (McKeague et al., 2003). For this reason, we adopted a stochastic time delay between 0 and 1 min for the beginning of necrosis in the sinusoid so that the necrosis process is non-deterministic.

**Figure 7:**
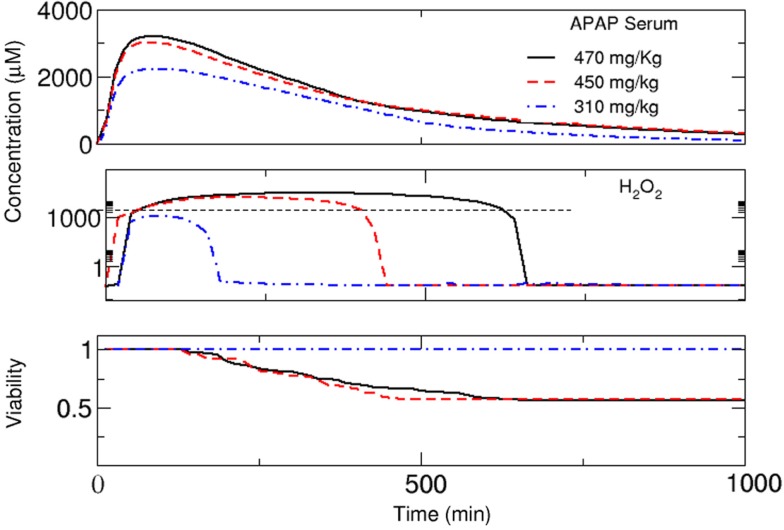
**Substance concentrations and mean viability in the lobule (see Figure 8) estimated using the multi-scale model**. Time courses are shown for the concentration of APAP in plasma, concentration of H_2_O_2_ in the cell (measured at the central vein, according to Figure 3), and total cell viability in the lobule for a dose of 310, 450, and 470 mg/kg, respectively. For viability estimation, it was assumed that an increase in H_2_O_2_ concentration above a critical threshold results in necrotic death of the respective cell.

This definition of necrosis together with our definition of clearance implies a continuous feedback between local cell behavior, clearance of the organ, and distribution of substances in the whole body. In order to find a region of critical APAP concentrations of interest, we performed several runs for different doses. We found that critical behavior is observed for doses above ∼300 mg/kg. In this region, we performed detailed simulations while assuming that the viability of the cells in the lobule remains constant below this dose. First, we performed simulations using an individual with a CYP3A4 activity of0.95 μmol/L_cell_/min and CYP2E1 activity of 1.0 μmol/Lcell/min and increased the dose from 310 to 470 mg/kg (Figure 7). For 310 mg/kg, no cell mortality was predicted. The selection of both CYP3A4 activities is in order to represent differences in the metabolism between women and men. The organ was able to perform detoxification and the toxic substance was completely eliminated after about 800 min. However, if the concentration is higher, cell mortality continues implying a gradual diminution of the capacity of the organ to eliminate APAP. Simultaneously, this leads to an increase in the APAP concentration in the liver (Figure 7). The slow elimination contributes to an increase in the intracellular level of H_2_O_2_ and to decreased cell viability in the organ. This is also relevant for repeated dose toxicity. In this example, a new dose larger than ∼300 mg/kg before ∼8 h after the initial dose could be lethal for this patient.

**Figure 8:**
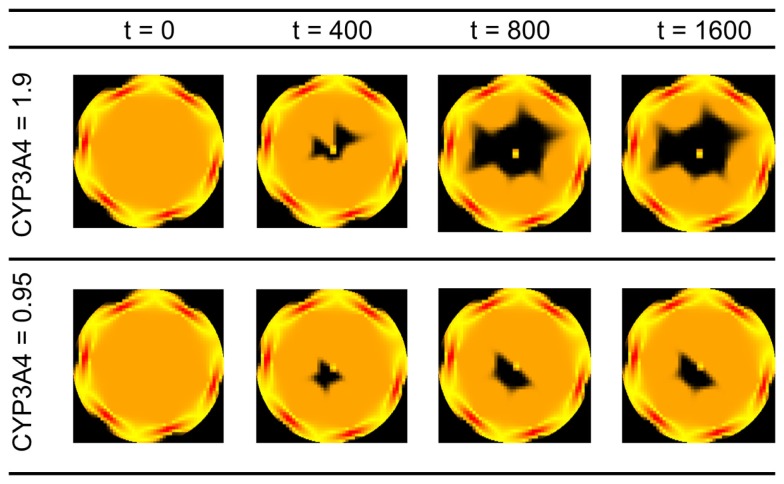
**Cell viability in the lobule at different time points for two different CYP3A4 activities after a single oral APAP dose of 393 mg/kg**. Viable cells are schematically illustrated n orange, dead cells in black. The spatial distribution corresponds to the detailed scheme and coordinates given in Figure 3. CYP activities in μM/min, time in min.

An advantage of kinetic models representing the dynamics at a cellular scale is the possibility to include inter-individual differences in gene expression or enzyme activities into the estimation of drug effects and toxicological risk. In the following, we will evaluate this exemplarily by considering selected variations in CYP enzyme activities (CYP3A4 activities of 0.95 and 1.9 μM/min and CYP2E1 activities of 1.0 and 5.0 μM/min (Bolt et al., 2003); the selection of the CYP2E1 is made in order to represent alcoholism and non-alcoholism) and by analyzing the distribution of substances as well as cell viability in the organ. The spatiotemporal estimation of cell viability in the liver lobule depending on the activity of CYP3A4 can be found in Figure 8. Given that the definition of the necrosis process is essentially non-deterministic, the degradation of the cells in the lobule is non-symmetric. We also observe that an increase in CYP3A4 activity strongly increases the risk of APAP-induced hepatotoxicity.

**Figure 9:**
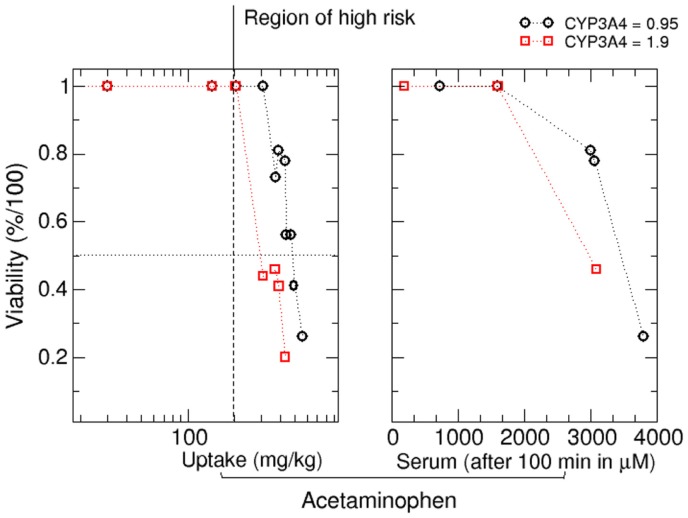
**Viability as a function of the oral APAP dose (left) and as a function of the APAP concentration in blood plasma at 33 h after APAP dose (right)**. Range of possible toxic daily APAP concentrations as reported in literature is given by the black dotted lines (see, e.g., the guideline suggested by Dart et al., 2006).

Based on this analysis, it is also possible to estimate critical doses for *in silico* patients from viability curves. Dose response curves at 2000 min after APAP exposure are given in Figure 9. Again, we observed a dose dependent and CYP3A4 expression dependent decrease in cell viability. Critical doses leading to a decrease in cell viability are in the range of values reported in literature (Schiødt et al., 1997; Larson et al., 2005). Acetaminophen induced hepatotoxicity has a large inter-individual variability and critical daily doses for individuals range between 10 and 1000 mg/kg [Larson et al., 2005; according to Dart et al., 2006 the acute grade of toxicity (or grade D) for patients of 6 years or older lies around 200 mg/Kg for acetaminophen].

**Figure 10:**
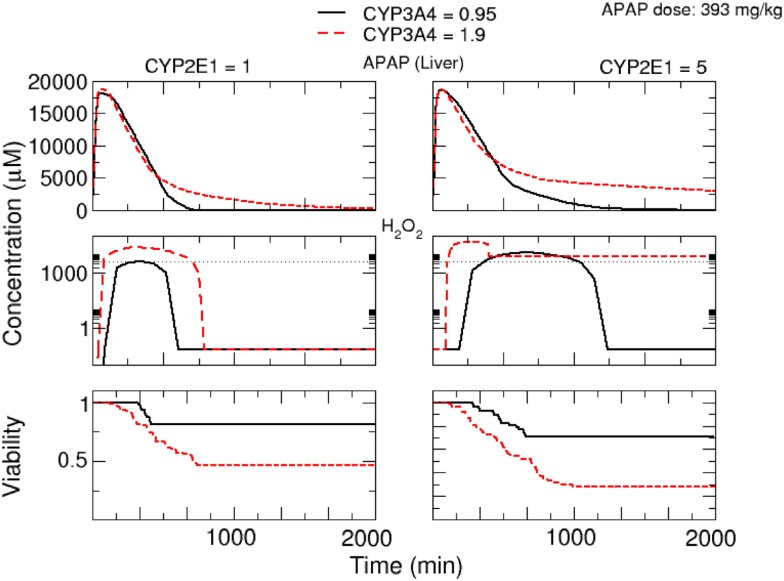
**Response upon oral APAP dose of 393 mg/kg depending on variations in the activity of CYP3A4 and CYP2E1**. Simulated concentrations of APAP in serum and of H_2_O_2_in cells at the central vein in the lobule are shown. The viability is the mean value of the whole lobule. The left group of panels corresponds to a CYP2E1 activity of 1 μmol/L_cell_/min, the right group of panels to a CYP2E1 activity of 5 μmol/L_cell_/min. Additionally, a comparison between two CYP3A4 activities, 0.95 and 1.9 μmol/L_cell_/min, is given in each figure.

Finally, we analyzed the time-dependent response upon APAP treatment influenced by variations in the activities of CYP2E1 and CYP3A4 (Figure 10). For both enzymes, a significant increase in APAP concentration in serum and H_2_O_2_ concentration in the liver was observed with increased enzyme activity leading to lower cell viability.

## Discussion

Russell and Burch (1959) recognized in their book “The principles of human experimental technique” the need to replace animal tests with other kinds of tests. Nowadays, numerous research initiatives are focusing on developing and improving methods and approaches suitable for reducing, refining, or even replacing tests on animals. Amongst different strategies, combinations of targeted *in vitro* experiments and *in silico* tools represent promising strategies for improved toxicity testing in the future (Basketter et al., 2012). In this study, we address this issue by focusing on a multi-scale modeling framework which integrates data derived from *in silico* as well as *in vitro* experiments. It is obvious that the strategy presented here has to be validated in other substances but it does bring research a further step forward toward predicting toxic effects *in vivo*. The development of novel computational technologies and their application to the modeling of human and animal physiology signify that *in silico* tests can, in fact, reduce and, in specific cases, even replace experimental tests on animals (Goldberg and Hartung, 2006).

A basic assumption underlying this study is that the primary toxic effect is mainly induced by cellular mechanisms. For this reason, we set-up and applied a model alternative with a reduced complexity of the liver, suitable for coupling hepatocytes and whole body. The integration of detailed cellular models into organs and a whole body model are basic differences compared with similar models (Wambaugh and Shah, 2010). Consequently, our model allows the analysis of local and systemic effects of parameters at different levels (cells, organ, and whole body).

As regards definition of the liver model, we were confronted with several alternatives and grades of complexity ranging, e.g., from detailed, structured liver sub-organelles (Wambaugh and Shah, 2010) over parallel tube (Ito and Houston, 2004) to well-stirred models (Kuepfer et al., 2012). Parallel tube models, which are a relatively simple approach, are best suited for intrinsic *in vitro* clearance, but not appropriate for *in vivo* analysis (Ito and Houston, 2004). In reality, there is a heterogeneous population of hepatocytes in the liver that has strong influence on the distribution and toxic effect of the compound on the cells, showing that a well-stirred model is clearly not appropriate for deriving robust *in vivo* predictions (Ito and Houston, 2004). In effect, we developed a model that combines a parallel tube model with dispersion, making it well-suited for the coupling of detailed cell mechanisms enabling (i) the implementation of individualized or stratified data on, e.g., gene expression and enzyme activities as well as (ii) the consideration of zonation. We believe that this approach is necessary for a better extrapolation of toxic effects from *in vitro* to *in vivo*. The possibility to define spatial distributions of substances and effects is a clear advantage over well-stirred or parallel tube representations (Kuepfer et al., 2012).

In this case study, we were able to predict the distribution of APAP and its metabolites in a whole body model and correlated concentrations of toxic metabolites with the onset of toxicity. Parameterization of the whole body model was performed using literature values and QSAR methods. Since we integrated a mechanistic, kinetic cellular model, we were able to analyze how individual properties – such as specific gene expression – affect pharmacokinetics and toxic outcomes. This is a clear difference compared with common PBPK approaches (Bois et al., 2010).

Concerning physiological parameters, such as body weight or associated volumes of specific internal organs, which are commonly considered in pharmacokinetic modeling (Price et al., 2003; Kuepfer et al., 2012), no variability was included in this study. Differences in these physiological parameters can be additionally introduced strongly increasing the complexity. Since our central aim was to show the effect of intracellular properties on drug metabolism, distribution of substances, and, eventually, organ-specific toxicity, we did not consider this aspect.

IVIVE-PBPK approaches were proposed for the extrapolation of toxic effects observed under *in vitro*–*in vivo* conditions (Rostami-Hodjegan, 2012). This approach can be useful not only for environmental risk assessment but also in drug development. Other new extrapolation techniques are based on a “middle out” approach which starts at the organ level and goes down to the cellular level in order to analyze, e.g., drug-induced liver injury (Howell et al., 2012).

The main difference between these methods and our multi-scale approach is that the method presented in this study allows the inclusion of physical parameters (such as blood flow, lobule size, diffusion, and dependence on temperature etc.) and important physiological properties, e.g., zonation of metabolic functions of organs and tissues. This means that the effects of organ structure and physiology on cellular function can be taken into account, aspects which can hardly be analyzed with a fore mentioned techniques.

An additional advantage is the possibility to analyze inter-individual variations in the toxicity of a compound, which is a major issue in pharmaceutical industry (Schiødt et al., 1997; Larson et al., 2005). Kinetic models of the mechanism of action of compounds are ideal for facing this issue as they can be used to predict inter-subject variability by including data, e.g., from well-characterized liver banks (Bucher et al., 2011; Riedmaier et al., 2011; Klein et al., 2012).

The modeling approach presented is based on a fairly complex network model of the mechanism of action for the compound of interest. This means that significant resources are required for reconstructing the network of each substance. Hence, this approach is suitable for a detailed characterization of substances. In this work, we performed a case study for one compound. Future work should focus on developing generic networks which can easily be transferred to new substances.

The presented liver model is essentially an average lobule assuming that the organ is of uniform tissue with a uniform blood flow and a rapid distribution of substances. In reality, the distribution of substances is non-homogeneous and time-dependent (Weiss et al., 2012), a fact that is exacerbated by drug toxicity or diseases such as cancer and cirrhosis. This aspect shows that a 3D representation of the organ would be a further step toward improving the predictivity of the liver model. In addition, the fact that drug metabolism is not only carried out in the liver requires the extension of this kind of mechanistic modeling to other organs.

In summary, we present a first multi-scale modeling approach integrating cellular and organ models in a whole body environment suitable for predicting spatiotemporal variations in drug response and toxicity. The presented work is an important basis for efficiently analyzing inter-individual differences upon drug treatment *in silico* and hence a significant step forward on the road to the development of *in silico* patients.

## Conflict of Interest Statement

The authors declare that the research was conducted in the absence of any commercial or financial relationships that could be construed as a potential conflict of interest.

## Supplementary Material

The Supplementary Material for this article can be found online at http://www.frontiersin.org/Drug_Metabolism_and_Transport/10.3389/fphar.2012.00204/abstract
